# Systematic Analysis of Intracellular Trafficking Motifs Located within the Cytoplasmic Domain of Simian Immunodeficiency Virus Glycoprotein gp41

**DOI:** 10.1371/journal.pone.0114753

**Published:** 2014-12-05

**Authors:** Thomas S. Postler, Jacqueline G. Bixby, Ronald C. Desrosiers, Eloísa Yuste

**Affiliations:** New England Primate Research Center, Department of Microbiology and Immunobiology, Harvard Medical School, Southborough, Massachusetts, United States of America; University of Pittsburgh, United States of America

## Abstract

Previous studies have shown that truncation of the cytoplasmic-domain sequences of the simian immunodeficiency virus (SIV) envelope glycoprotein (Env) just prior to a potential intracellular-trafficking signal of the sequence YIHF can strongly increase Env protein expression on the cell surface, Env incorporation into virions and, at least in some contexts, virion infectivity. Here, all 12 potential intracellular-trafficking motifs (YXXΦ or LL/LI/IL) in the gp41 cytoplasmic domain (gp41CD) of SIVmac239 were analyzed by systematic mutagenesis. One single and 7 sequential combination mutants in this cytoplasmic domain were characterized. Cell-surface levels of Env were not significantly affected by any of the mutations. Most combination mutations resulted in moderate 3- to 8-fold increases in Env incorporation into virions. However, mutation of all 12 potential sites actually decreased Env incorporation into virions. Variant forms with 11 or 12 mutated sites exhibited 3-fold lower levels of inherent infectivity, while none of the other single or combination mutations that were studied significantly affected the inherent infectivity of SIVmac239. These minor effects of mutations in trafficking motifs form a stark contrast to the strong increases in cell-surface expression and Env incorporation which have previously been reported for large truncations of gp41CD. Surprisingly, mutation of potential trafficking motifs in gp41CD of SIVmac316, which differs by only one residue from gp41CD of SIVmac239, effectively recapitulated the increases in Env incorporation into virions observed with gp41CD truncations. Our results indicate that increases in Env surface expression and virion incorporation associated with truncation of SIVmac239 gp41CD are not fully explained by loss of consensus trafficking motifs.

## Introduction

The envelope glycoproteins (Env) of the human immunodeficiency virus (HIV) and its close relative the simian immunodeficiency virus (SIV) consist of two components, the surface subunit gp120 (SU) and the transmembrane subunit gp41 (TM) [1]–[3]. gp120 and gp41 derive from a common precursor protein, gp160, through proteolytic cleavage in the Golgi apparatus [4]–[6]. After cleavage, gp120 and gp41 remain noncovalently associated and are transported through the secretory pathway to the cell membrane where gp41 anchors the complex in the membrane while gp120 is located entirely outside of the cell [7], [8]. Once at the cell membrane, Env complexes are quickly internalized by clathrin-mediated endocytosis, resulting in a very low steady-state level of Env cell-surface expression [9]–[12]. Two synergistically acting, highly conserved endocytosis motifs have been identified in the sequence of the cytoplasmic domain of gp41 (gp41CD): a membrane-proximal YXXΦ motif [10]–[12] and a C-terminal dileucine motif [13]. The YXXΦ motif, where X is any and Φ is a hydrophobic amino acid, interacts with the µ2 subunit of the clathrin-associated adaptor protein complex AP-2 to effect endocytosis [9], [14]–[16]. The presence of this sequence contributes to SIV virulence since animals infected with mutants lacking the motif display reduced viral loads and delayed progression to AIDS compared to infection with wild-type virus [17], [18]. The C-terminal dileucine motif, whose functionality has only been conclusively demonstrated in HIV-1 Env, is believed to also bind to the AP-2 complex [13], [14], although evidence for this remains indirect. The AP-2 complex is thought to typically interact with dileucine motifs through its σ2 subunit [19], but an interaction between σ2 and gp41CD has not yet been demonstrated. Interestingly, the C-terminal dileucine motif of gp41CD is not entirely consistent with the consensus sequence for clathrin-dependent endocytosis, neither mediated by the AP-2 complex (motif [D/E]XXXL[L/I/M]) nor by the Golgi-localized, γ-ear-containing, ARF-binding (GGA) family of adaptor proteins (motif DXXLL) [19], [20]. It is worth noting that not all dileucine motifs constitute trafficking signals. The third conventional endocytosis motif, FXNPXY, is not represented in gp41CD.

Truncation of SIV gp41CD downstream of the membrane-proximal YXXΦ motif strongly increases cell-surface levels of Env [11], [14], [21]–[23]. This increase, however, is considerably greater than the modest increase resulting from mutation of the C-terminal dileucine motif alone, implying that gp41CD may contain additional endocytosis motifs [13]. However, attempts to define such motifs have for the most part not been successful [13], [15], [24].

Both the membrane-proximal YXXΦ motif and the C-terminal dileucine motif have been shown to interact not only with AP-2 but also with the AP-1 complex [9], [11], [14]–[16], [25], which enables clathrin-mediated trafficking between the *trans*-Golgi network (TGN) and endosomes as well as basolateral sorting [26]. This interaction between AP-1 and gp41CD is thought to be relevant for direct cell-to-cell viral transmission [27]–[29].

In addition to these two established trafficking motifs, gp41CD of SIVmac includes three other YXXΦ motifs (only one other in HIV-1 Env) and four L[L/I] motifs (six in HIV-1 Env), as well as three IL motifs [22], [30]. A recent study by Bhakta *et al*. systematically mutated the YXXΦ motifs and the dileucine motifs in gp41CD of HIV-1 and reported a progressive loss of fusogenicity and infectivity with accumulation of mutations but little effect on cell-surface expression of Env [24]. We have carried out a similar study in parallel with Env of SIVmac239 and SIVmac316 (a macrophage-tropic, neutralization-sensitive derivate of SIVmac239 [31]) to assess the effects of systematic mutation on cell-surface expression, virion incorporation and viral infectivity. The motifs are well conserved in number and in location between the gp41CDs of HIV-1 and SIVmac. Our results with SIVmac contrast with the results reported for HIV-1 Env [24] as progressive mutations of the N-terminal potential trafficking motifs led to a moderate increase in Env incorporation, rather than a decrease. However, none of the mutations of potential intracellular-trafficking motifs in gp41CD of SIVmac239 approximated the strong effects observed with truncations of gp41CD, suggesting that the effects of truncation are not simply due to the loss of these specific sequence motifs. In contrast, we found that Env of SIVmac316 is remarkably more responsive to these mutations than SIVmac239 Env, despite the fact that its gp41CD sequence differs from that of SIVmac239 by only one amino acid (R751G) [31].

## Materials and Methods

### Plasmids and Site-Directed Mutagenesis

Mutations were first introduced into plasmid p239SpE3′ *nef* open, which encodes the 3′ half of the SIVmac239 genome [32], [33]. Site-directed mutagenesis was performed with the QuikChange II kit (Agilent Technologies, Santa Clara, CA, U.S.A.), following the manufacturer's recommendations. Primers were synthesized by Sigma-Aldrich (St. Louis, MO, U.S.A.). All mutations except E767* were selected to preserve the amino-acid sequence of the overlapping ORFs of *tat*, *rev* and *nef*. Mutation E767* did alter the second exon of *rev* by changing an UGA Arg codon to an AUA Ile codon and the second exon of *tat* by changing a UAG stop codon to a Tyr codon adding six amino acids at the end of Tat (YNIPIS). To produce full-length infectious virions, p239SpE3′ *nef* open and derivative plasmids were ligated to plasmid p239SpSp5′ [32] and transfected into HEK293T cells (ATCC, Manassas, VA, U.S.A.) as described below. For transient Env expression, mutations were cloned *via* the *Sph*I and *EcoR*I restrictions sites from p239SpE3′ *nef* open into plasmid pSIVΔgpV, which encodes the genome of SIVmac239 with a major deletion in the *gag-pol* region [34]. This construct expresses Env under control of the viral long terminal repeat (LTR), as well as the viral proteins Vpx, Vpr, Tat, Rev and Nef, but it does not produce virions. All mutations were verified by DNA sequencing.

### Cell culture, transfection and virus stocks

HEK293T cells were maintained in Dulbecco's modified Eagle medium (DMEM) supplemented with 10% fetal bovine serum, 25 mM HEPES, 2 mM L-glutamine, 100 units/ml penicillin, and 100 µg/ml streptomycin (all purchased from Life Technologies, Grand Island, NY, U.S.A.). C8166-45 SIV-SEAP cells [35] were cultivated in RPMI 1640 medium supplemented with 10% fetal bovine serum, 25 mM HEPES, 2 mM L-glutamine, 100 units/ml penicillin, and 100 µg/ml streptomycin. Cells were transfected with the Calcium Phosphate Method using the ProFection Mammalian Transfection System (Promega, Madison, WI, U.S.A.). To generate full-length provirus and produce infectious virions, 3 µg of plasmid p239SpE3′ *nef* open or derivatives and 3 µg of its counterpart encoding the 5′ half of the SIVmac239 genome, p239SpSp5′ [32], were each digested with restriction enzyme *Sph*I (New England BioLabs, Ipswich, MA, U.S.A.) and subsequently ligated together using T4 DNA ligase (Promega, Madison, WI, U.S.A.). Ligated constructs were subsequently used to transfect HEK293T cells with the Calcium Phosphate Method. Medium was changed on day 2 after transfection and supernatant harvested on day 3. Virus content was assessed based on the concentration of p27 capsid protein in the supernatant, as determined by antigen-capture assay (Beckman Coulter, Indianapolis, IN, U.S.A.).

### Antibodies and Western blot

The following primary antibodies were used in this study: rhesus α-gp120 antibody 1.9C [36]; rhesus α-gp120 antibody 3.11H [37]; mouse α-p27 antibody 2F12 (NIH AIDS Reagent Program) [38]; and rabbit α-β-Tubulin (9F3) (Cell Signaling Technology, Danvers, MA, U.S.A.). Secondary antibodies conjugated to horse-radish peroxidase (HRP) were purchased from Santa Cruz Biotechnology (Dallas, TX, U.S.A.). For Western blots of whole-cell lysates, cells were lysed with NP-40 Lysis Buffer (50 mM HEPES pH 7.4, 150 mM NaCl, 1% NP-40 in H_2_O) with Complete protease inhibitor cocktail (Roche Applied Science, Indianapolis, IN, U.S.A.) by vigorous vortexing. Cellular debris was pelleted by centrifugation, supernatants were transferred to fresh vials and mixed with 2X Laemmli Buffer (Sigma-Aldrich, St. Louis, MO, U.S.A.). Protein concentrations were determined by BCA assay (Thermo Fisher Scientific, Rockford, IL, U.S.A.). Equivalent amounts of protein were separated by SDS-PAGE and blotted onto polyvinylidene fluoride membranes (Millipore, Billerica, MA, U.S.A.). Unspecific antibody binding was blocked by incubation with 5% non-fat dry milk (NFDM) in phosphate-buffered saline (PBS) with 0.05% TWEEN 20. After incubation with primary and secondary antibodies, membranes were incubated with chemiluminescence substrate (Thermo Fisher Scientific, Rockford, IL, U.S.A.) and signal detected by a phosphorimager (FujiFilms, Edison, NJ, U.S.A.).

### Flow cytometry

Cell-surface expression of Env was determined by flow cytometry in cells transfected with pSIVΔgpV constructs (see above). HEK293T cells were transiently co-transfected with pSIVΔgpV or its mutated derivatives and the GFP expression plasmid pTracer (Life Technologies, Grand Island, NY, U.S.A.), as described above. Three days after transfection, cells were harvested, washed twice with 2% fetal bovine serum in PBS and incubated with rhesus α-gp120 antibody 1.9C [36]. Cells were then incubated with phycoerythrin-conjugated or allophycocyanin-conjugated α-human immunoglobulin G antibody (Jackson ImmunoResearch Laboratories, West Grove, PA, U.S.A.) and subsequently fixed in 2% formaldehyde in PBS before flow cytometry. Data were analyzed using FlowJo software version 6.4.1. (Tree Star, Ashland, OR, U.S.A.). Cellular debris and GFP-negative events were excluded from analysis.

### Env incorporation assay

Env incorporation into virions was assessed as described previously [23], [39]. Initial clarification of virus-containing supernatants was conducted by two consecutive spins at 3,000 rpm for 10 min. Subsequently, virus was pelleted by centrifugation at 13,000 rpm and 4°C for 2 h. The resulting viral pellet was resuspended in 1 ml PBS and pelleted a second time as above. The pellet was then resuspended in 50 µl PBS and the amount of p27 quantified by antigen-capture assay (Beckman Coulter, Indianapolis, IN, U.S.A.). Equivalent amounts of virus, as assessed by p27 content, were mixed with Laemmli Buffer and heated in boiling water for 4 min. Samples were then separated by SDS-PAGE and blotted onto polyvinylidene fluoride membranes (Millipore, Billerica, MA, U.S.A.). After blocking membranes with 5% NFDM in PBS with 0.05% TWEEN 20, gp120 and p27 were detected with antibodies 3.11H and 2F12, respectively. After incubation with corresponding HRP-coupled secondary antibodies, membranes were incubated with chemiluminescence substrate (Thermo Fisher Scientific, Rockford, IL, U.S.A.) and bands detected with a phosphorimager (FujiFilms, Edison, NJ, U.S.A.). Band intensity was quantified using ImageGauge software version 4.22 (FujiFilms, Edison, NJ, U.S.A.).

### Infectivity assay

Infectivity of virus mutants was assessed using C8166-45 SIV-SEAP cells [35], [39]. On 96-well plates, triplicate sets of seven or nine 2-fold dilutions were prepared. Each row also contained a well with medium without virus. To each well, 5⋅10^3^ C8166-45 SIV-SEAP cells were added and the plate was transferred to a humidified CO_2_ incubator maintained at 37°C. On day 3 after infection, activity of secreted alkaline phosphatase (SEAP) was measured with the Phospha-Light kit (Life Technologies, Grand Island, NY, U.S.A.). Linear regression analysis of data points was used to normalize SEAP activity to the amount of input virus (SEAP/ng) for each dilution series.

## Results

### Mutation of potential intracellular-trafficking motifs in gp41CD

**Figure 1:**
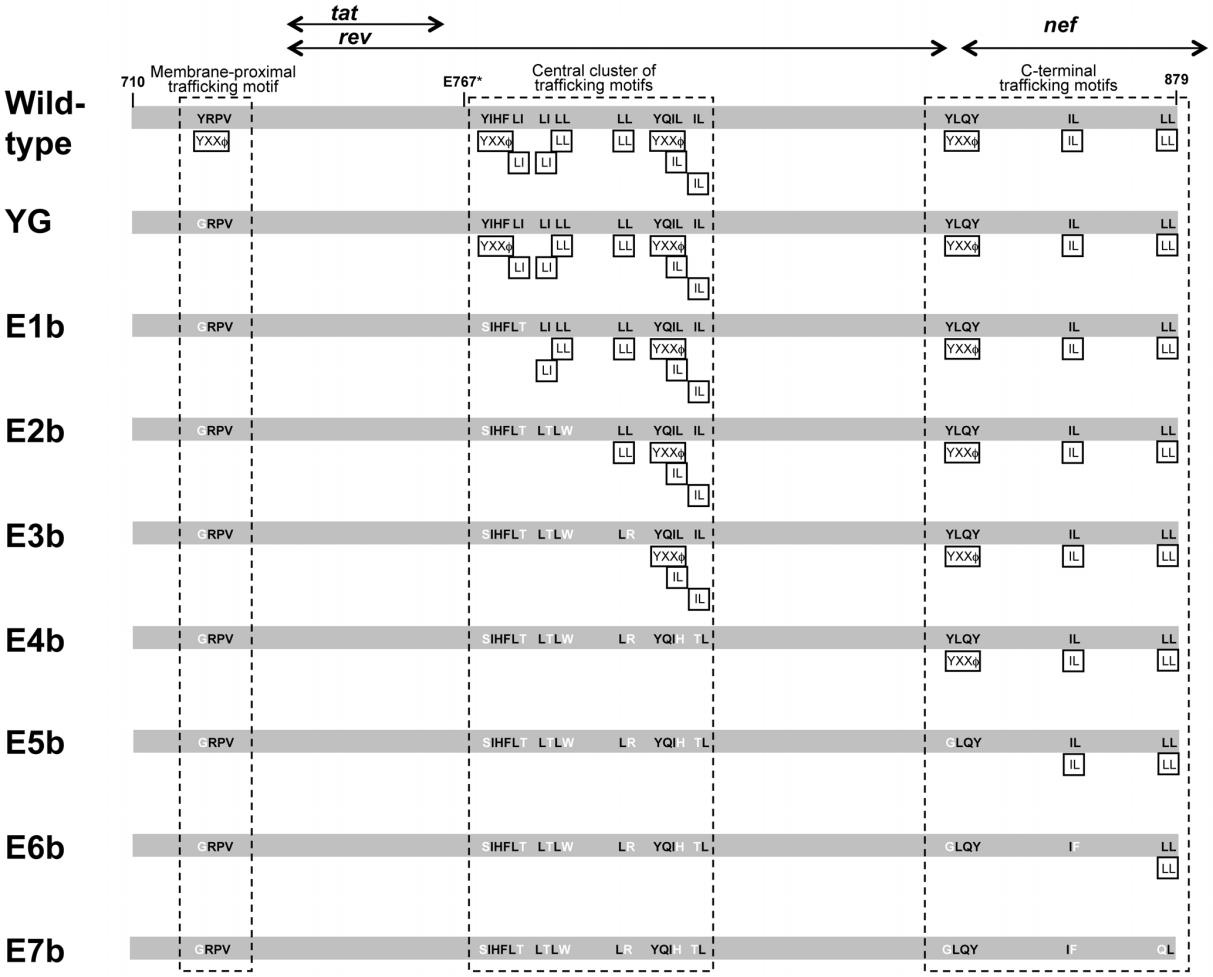
Schematic representation of mutations within the cytoplasmic domain of gp41. Black boxes indicate location and type of potential trafficking motifs. Black arrows represent overlapping reading frames for *rev*, *tat*, and *nef*, respectively. Stripes mark the membrane-proximal trafficking motif, the central cluster of trafficking motifs and the C-terminal trafficking motifs.

In order to assess the contribution of specific sequences in gp41CD to intracellular trafficking of Env in a completely unbiased manner, we introduced mutations to eliminate all twelve YXXΦ and dileucine motifs in SIV gp41CD (Fig. 1), regardless of the adjacent sequences. A previous study by Bowers *et al*. has shown that separate mutation of nine of these trafficking motifs individually, all located C-terminally of the membrane-proximal YXXΦ motif, did not alter cell-surface expression levels of Env to the same degree as truncation C-terminal of the membrane-proximal YXXΦ motif, raising the possibility of functional redundancy among these motifs [15]. We therefore chose to mutate combinations of these potential trafficking motifs, including a combination of all twelve, to ensure that any functional contribution would not be masked by redundant elements. A total of seven combination mutants and one single-amino-acid mutant were initially generated in the genetic context of the molecular clone SIVmac239. Mutations were selected to ensure that the amino-acid sequences of Tat, Rev and Nef, whose ORFs partly overlap with the *env* reading frame, remained unchanged. We also included an Env mutant with a truncation at amino acid E767 (E767*), which is located immediately N-terminal of the second potential trafficking motif (Fig. 1). This truncation has been previously associated with increased incorporation into virions, increased cell-surface expression and increased infectivity [23].

**Figure 2:**
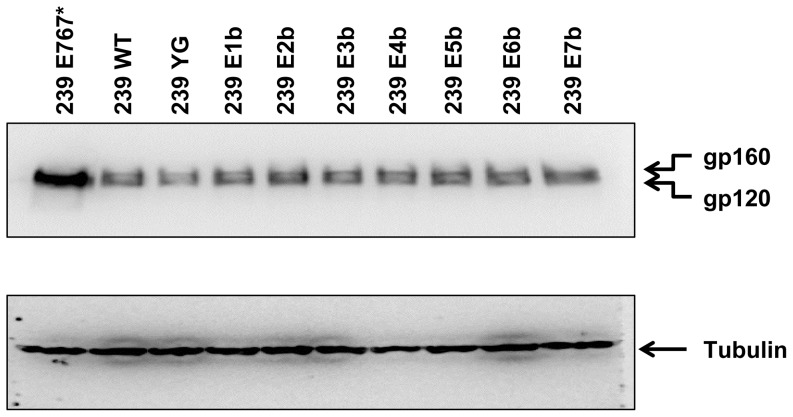
Point mutations of potential trafficking motifs do not affect cellular expression levels of SIVmac239 Env. HEK293T cells were transfected with SIVmac239 Env mutants. On day 3 after transfection, cells were lysed and equivalent amounts of lysate analyzed by Western blot. gp120 and gp160 were detected by antibody 3.11H. Tubulin was used as input control. WT, wild-type; E767*, Env mutant with truncation at amino acid E767.

To test whether any of these mutations affected protein stability or Env cleavage, all nine mutant proteins were transiently expressed in HEK293T cells by DNA transfection and lysates were analyzed by Western blot. We used the Env expression cassette pSIVΔgpV [34], which is derived from the full-length molecular clone of SIVmac239 with major deletions in the *gag-pol* region and expresses Env under control of the LTR promoter, rather than a highly expressing codon-optimized Env plasmid, to avoid possible complications from non-physiologically high levels of Env produced by the transfected cell or from the absence of Nef, which has been shown to interact with the trafficking machinery of the host cell [40]. Expression levels of all Env variants with amino-acid substitutions were comparable to the wild-type protein and cleavage of gp160 into gp120 and gp41 was not impaired (Fig. 2). Intriguingly, the truncated Env mutant E767* exhibited much higher levels of protein in whole-cell lysates, indicating that removing the C-terminal 113 amino acids from gp41CD drastically enhances protein expression and/or stability.

### Cell-surface expression of Env mutants

**Figure 3:**
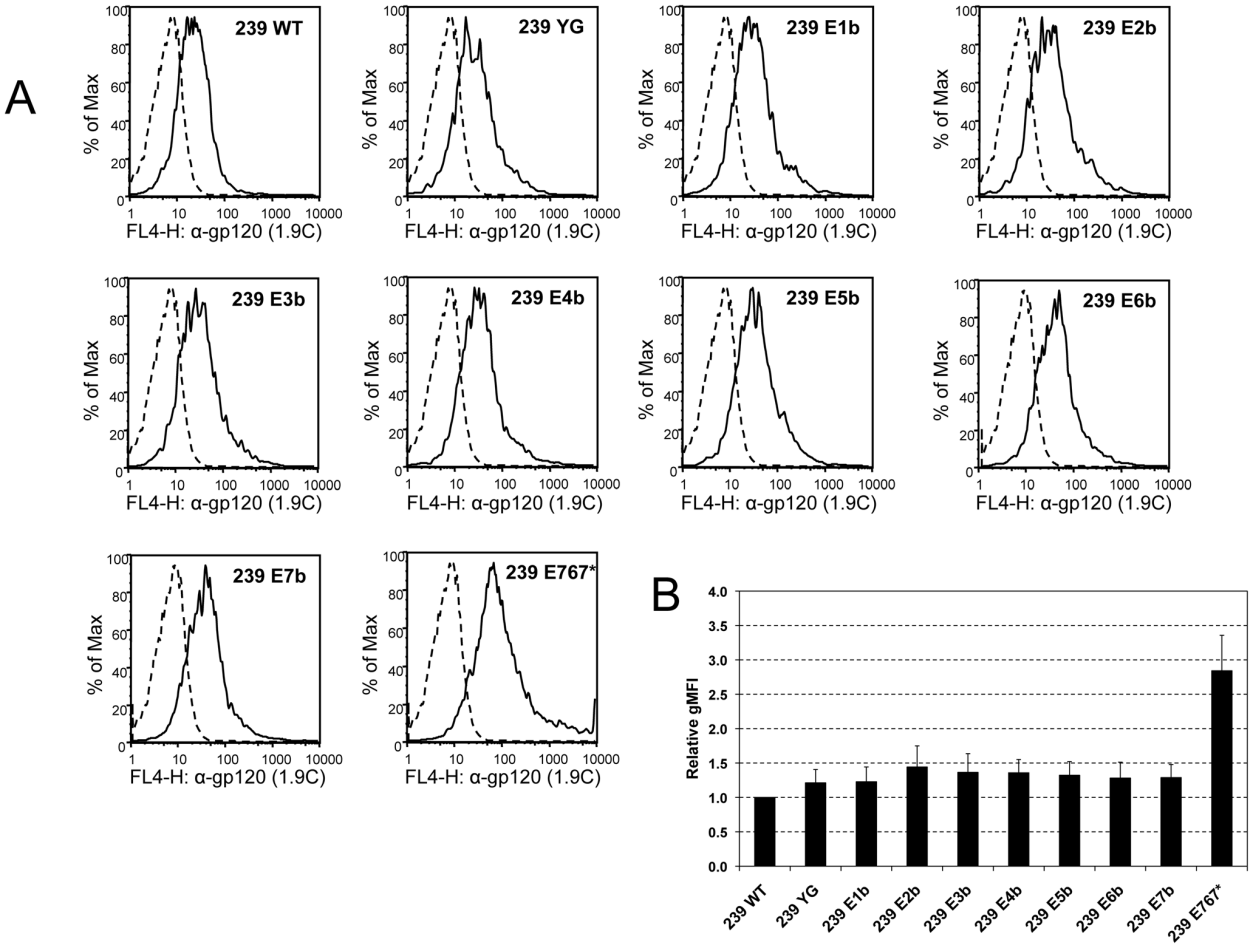
Cell-surface expression of SIVmac239 Env is not significantly altered by mutation of potential trafficking motifs. HEK293T cells were transiently co-transfected with SIVmac239 Env mutants and a GFP expression plasmid. On day 3 after transfection, cells were fixed and stained with anti-gp120 antibody 1.9C. Only the GFP-positive population was included in the analysis of Env expression. WT, wild-type; E767*, Env mutant with truncation at amino acid E767. (**A**) Histograms from a representative experiment. Solid line represents cells co-transfected with Env and GFP, dashed line shows negative controls (transfected with GFP only). (**B**) Relative geometric mean fluorescence intensity (gMFI), normalized for wild-type Env (239 WT). Error bars indicate standard deviation of four independent experiments.

The rapid internalization of wild-type Env from the cell surface by clathrin-mediated endocytosis cannot be fully explained by the activity of the membrane-proximal YXXΦ and the C-terminal dileucine motifs alone [13]–[15], [23]. To address the possibility of additional, redundantly functioning endocytosis motifs in SIV gp41CD, we tested the levels of cell-surface expression of the Env mutants by flow cytometry (Fig. 3 and Table 1). While there was a consistent trend towards slightly higher levels of cell-surface expression for all eight mutants, none of these increases reached statistical significance. The greatest amount of Env was detected on the cell surface when the first five N-terminal motifs were mutated (mutant E2b), at levels merely 1.4-fold higher than observed for wild-type Env (compared to a 1.2-fold increase with mutant YG).

**Table 1:** Summary of phenotypes associated with mutation of potential trafficking motifs in gp41CD.

Envelope	Cell-Surface Expression^a^	Virion Incorporation^a^	Infectivity^a^
**SIVmac239**			
Wild-type	100	100	100
YG	121	365	72
E1b	123	n.q.	127
E2b	144	n.q.	126
E3b	136	n.q.	138
E4b	136	820	111
E5b	132	n.q.	91
E6b	128	93	32
E7b	129	39	29
E767*	284	2500^b^	250^b^
**SIVmac316**			
Wild-type	n.d.	n.q.	100
YG	n.d.	n.q.	129
E3b	n.d.	n.q.	723
E7b	n.d.	n.q.	22
E767*	n.d.	1400^b^	48000^b^

a Presented as percentage of homologous wild-type Env.

b Data published in [39]. N.d., not determined. N.q., not quantified.

We confirmed that truncation of Env at amino acid E767, which removes all potential trafficking motifs except the membrane-proximal YXXΦ, led to a major increase in Env cell-surface expression (2.8-fold above wild-type levels) [23].

### Incorporation of mutant Env into virions

**Figure 4:**
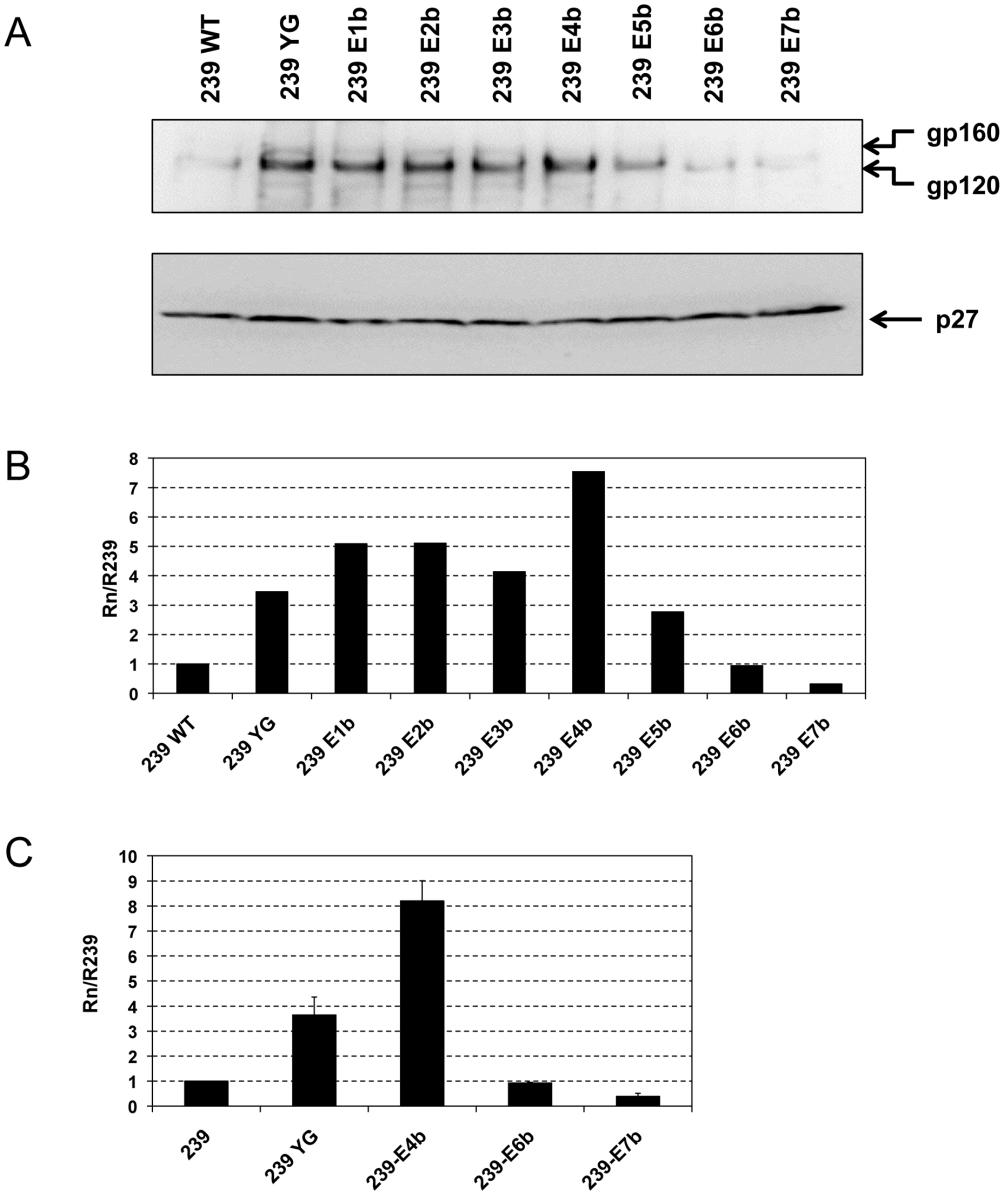
Mutation of potential trafficking motifs affects virion incorporation of SIVmac239 Env. Viruses were produced by transfection of HEK293T cells and virions were pelleted from the clarified supernatants. (**A**) gp160/gp120 (top panel) and p27 (bottom panel) were detected by Western blot using antibody 3.11H and 2F12, respectively. (**B**) Relative Env incorporation into virions. The ratios of gp120 to p27 were calculated by phosphorimaging analysis. Data are presented as the gp120:p27 ratio of mutant virions (Rn) relative to the gp120:p27 ratio of SIVmac239 wild-type (WT) virions (R239). (C) Mutants YG, E4b, E6b and E7b were quantified in triplicate. Shown is the average of independent experiments, error bars indicate standard deviation.

Next, we tested the extent to which mutation of the potential trafficking motifs in gp41CD affected incorporation of SIVmac239 Env into virions. To this end, infectious virus was produced in HEK293T cells and pelleted from clarified supernatant. Virion samples normalized for p27 content were then subjected to analysis by Western blot (Fig. 4A) and the ratio of Env to p27 in mutant virions relative to wild-type virions was determined by densitometry (Fig. 4B, C and Table 1). In stark contrast to HIV-1 Env, whose incorporation has been reported to be strongly reduced in mutants of potential gp41CD trafficking motifs [24], most mutant forms of SIV Env were incorporated into virions at higher levels than wild-type Env. Mutation of the membrane-proximal YXXΦ motif alone increased Env incorporation by 3.7-fold, as reported previously [23]. Disruption of the next eight potential trafficking motifs (mutants E1b – E4b), which cluster around the center of gp41CD, slightly enhanced Env incorporation further, with mutant E4b showing the highest level at 8.2-fold more Env than wild-type virions. Further mutations reduced Env incorporation in a step-wise fashion. Mutant E6b, which retains only the C-terminal dileucine motif, incorporated Env at levels comparable to wild-type virus, whereas mutant E7b, lacking all potential trafficking motifs, incorporated Env at merely 39% of wild-type virus. While not specifically included in parallel in these experiments, previous results from our laboratory have demonstrated a 25-fold increase in Env incorporation into SIVmac239 virions with the E767* truncation [23], [39].

Although the membrane-proximal YXXΦ motif clearly plays a dominant role in reducing the amount of Env incorporated into SIV virions, these data suggest that the potential trafficking motifs in the center of gp41CD also modulate SIVmac239 Env incorporation to some extent. Furthermore, these results indicate that the C-terminal cluster of trafficking motifs, in particular the C-terminal dileucine motif, may actively contribute to efficient incorporation of Env into virions.

### Relevance of potential trafficking motifs for viral infectivity of SIVmac239

**Figure 5:**
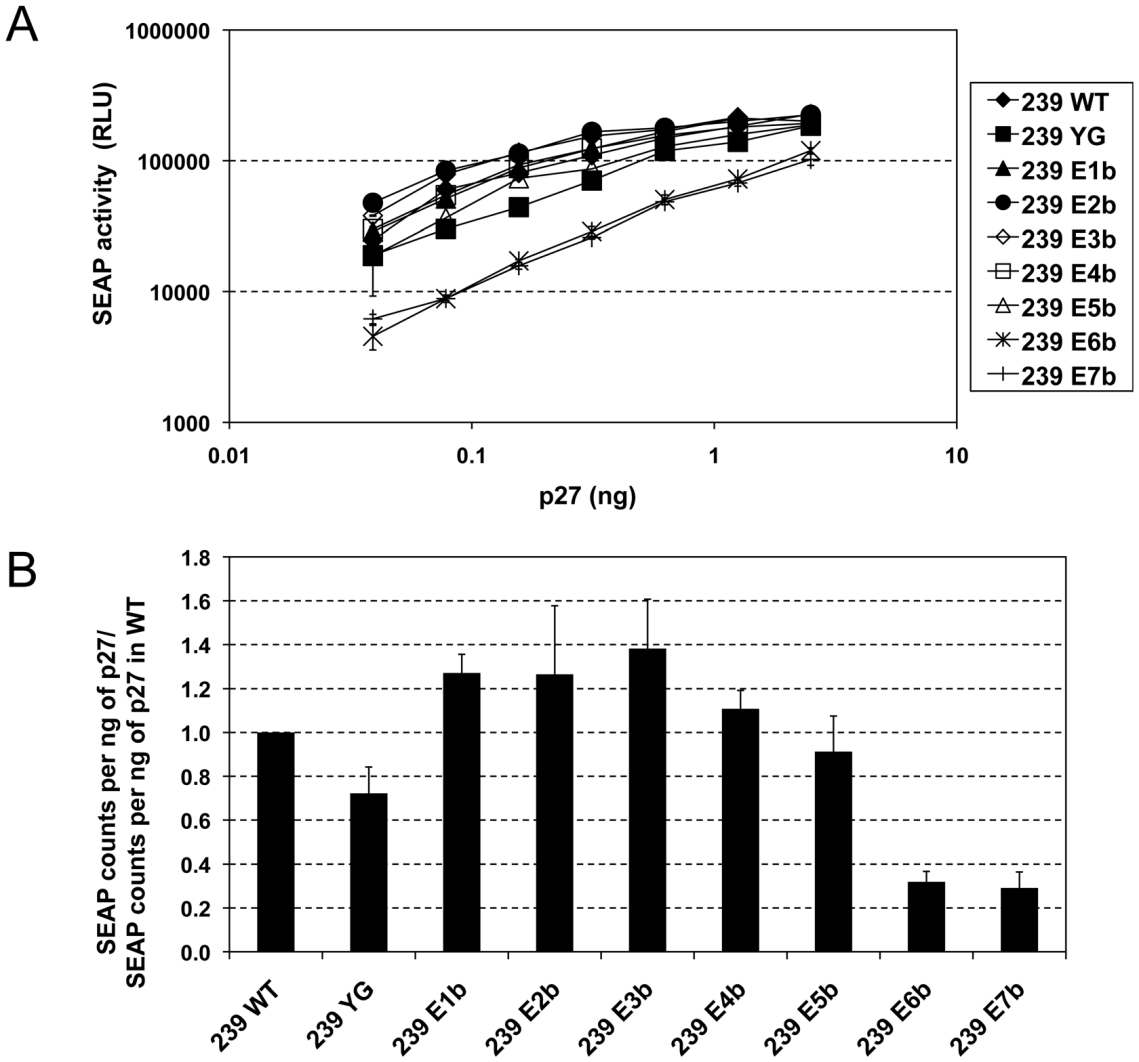
Viral infectivity of SIVmac239 is not affected by mutation of most potential trafficking motifs in gp41CD. Viruses were produced by transfection of HEK293T cells and virions were pelleted from the clarified supernatants. Stocks were normalized for p27 content and used to infect C8166-45 SIV-SEAP cells with serial dilutions. (**A**) SEAP activity 3 days after infection. Error bars indicate standard deviation of triplicates. (**B**) SEAP activity per ng of p27 input, normalized for wild-type virus. Average of 2–3 independent experiments, error bars indicate standard deviation. WT, wild-type. SEAP, secreted alkaline phosphatase.

To assess the relevance of the potential trafficking motifs in gp41CD for viral infectivity, we infected C8166-45 SIV-SEAP cells with mutant virus produced in HEK293T cells and normalized for p27 content. C8166-45 SIV-SEAP cells express secreted alkaline phosphatase (SEAP) upon infection with SIV; SEAP levels in the supernatant can be conveniently detected by a chemiluminescence-based assay and are directly proportional to the amount of virus infecting the cells [35]. Mutation of most potential trafficking motifs in gp41CD did not have major effects on viral infectivity (Fig. 5 and Table 1, mutants YG and E1b – E5b). In a previous study, we have demonstrated that even a 25-fold increase in Env incorporation increases infectivity of SIVmac239 by only 2.5-fold [23]. Therefore, it is to be expected that the observed increase in Env incorporation of up to 8.2-fold (Fig. 4 and Table 1) would not translate into a drastic change in viral infectivity. Eliminating 11 or 12 of the motifs, however, reduced infectivity by 3-fold compared to wild-type virus (Fig. 5 and Table 1, mutants E6b and E7b). Interestingly, mutant E6b retained near-wild-type levels of Env incorporation into virions; thus, the reduction in viral infectivity cannot be attributed to a reduction in Env incorporation, but more likely results from some other structure/function defect.

### Role of the membrane-proximal YXXΦ motif

**Figure 6:**
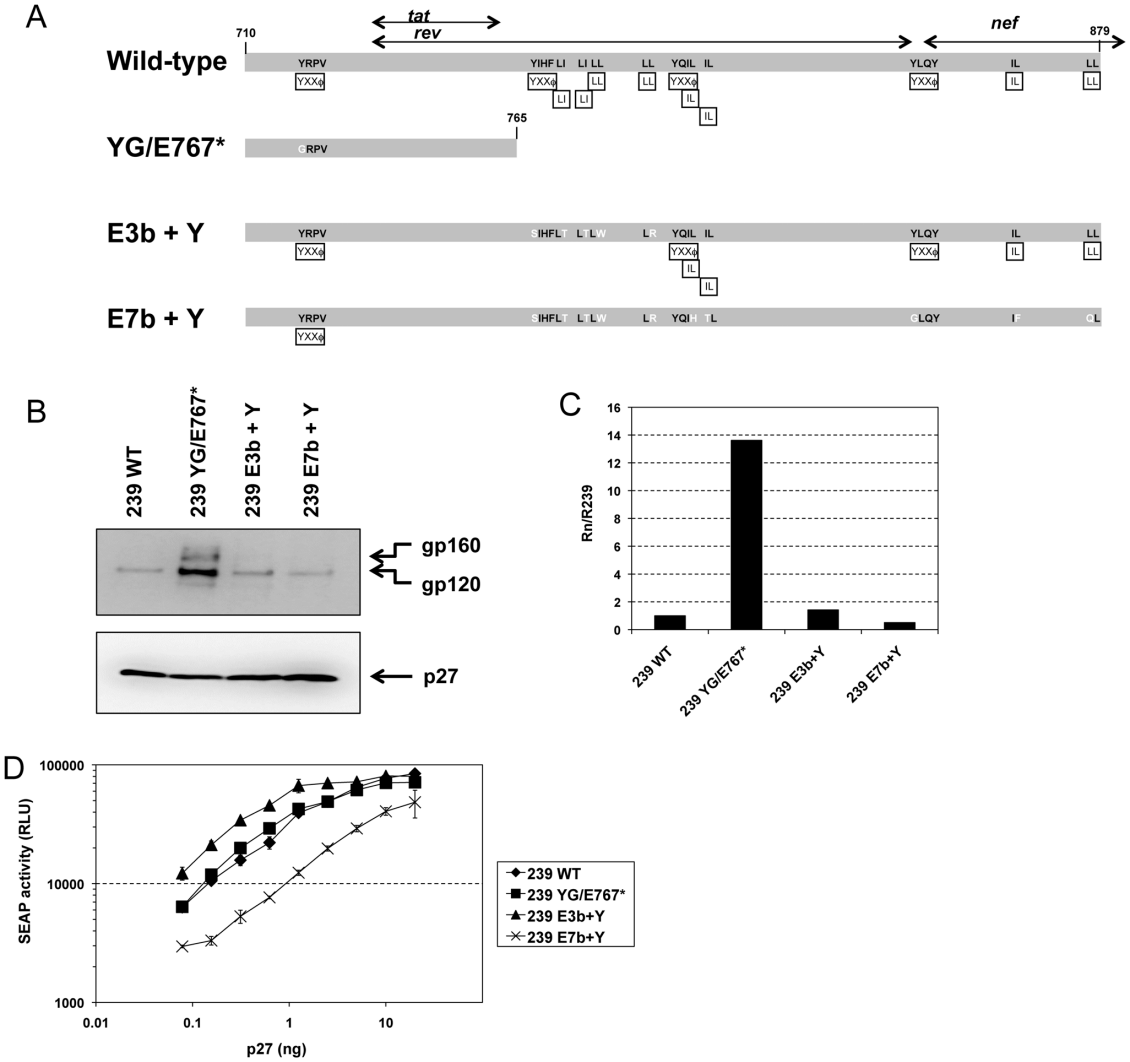
Role of the membrane-proximal YXXΦ motif. (**A**) Schematic representation of additional mutations within the cytoplasmic domain of gp41. Black boxes indicate location and type of potential trafficking motifs. Black arrows represent overlapping reading frames for *rev*, *tat*, and *nef*, respectively. (**B and C**) Incorporation of mutant Env into virions. Viruses were produced by transfection of HEK293T cells and virions were pelleted from the clarified supernatants. (B) gp160/gp120 (top panel) and p27 (bottom panel) were detected by Western blot using antibody 3.11H and 2F12, respectively. (C) Relative Env incorporation into virions. The ratios of gp120 to p27 were calculated by phosphorimaging analysis. Data are presented as the gp120:p27 ratio of mutant virions (Rn) relative to the gp120:p27 ratio of SIVmac239 wild-type (WT) virions (R239). (**D**) Infectivity of mutant viruses. Stocks were normalized for p27 content and used to infect C8166-45 SIV-SEAP cells with serial dilutions. SEAP activity 3 days after infection. Error bars indicate standard deviation of triplicates.

To probe further the contributions of the membrane-proximal YXXΦ motif to the observed phenotype, we reconstituted this motif in SIVmac239 mutants E3b (E3b + Y) and E7b (E7b + Y). Additionally, we created a mutant with a truncation at Env amino acid E767 without the membrane-proximal YXXΦ motif (YG/E767*) (Fig. 6A). Env incorporation into virions (Fig. 6B, C) and infectivity (Fig. 6D) of these mutants were assessed. As expected, removal of all potential trafficking motifs in gp41CD by truncation at amino acid E767* in conjunction with elimination of the membrane-proximal YXXΦ motif (mutant YG/E767*) led to a strong increase in Env incorporation into virions (Fig. 6B, C). Interestingly, we did not detect a corresponding increase in viral infectivity of mutant YG/E767* (Fig. 6D). This phenotype may be explained by reports demonstrating that loss of the membrane-proximal YXXΦ motif of HIV-1 Env considerably reduces viral infectivity [24], [41], although the importance of this motif for infectivity of SIV appears to be far less pronounced as its loss alone reduced SIV infectivity only slightly (Fig. 5, Table 1 and [23]). Nonetheless, any increase in infectivity conveyed by the E767* mutation may be offset by the reduction in infectivity associated with the loss of the membrane proximal YXXΦ motif. Reconstitution of the membrane-proximal YXXΦ motif did not affect Env incorporation in the context of the E3b + Y mutant (Fig. 6B, C). It did, however, result in a detectable increase in viral infectivity compared to wild-type SIVmac239 (Fig. 6D). This further supports the notion that the membrane-proximal YXXΦ motif modulates SIV infectivity at least in part through a mechanism independent of Env incorporation into virions. In contrast, reintroduction of the membrane-proximal YXXΦ motif did not rescue the reduced levels of Env incorporation and viral infectivity of mutant E7b (Fig. 6B, C and D).

### Env incorporation in the genetic context of SIVmac316

**Figure 7:**
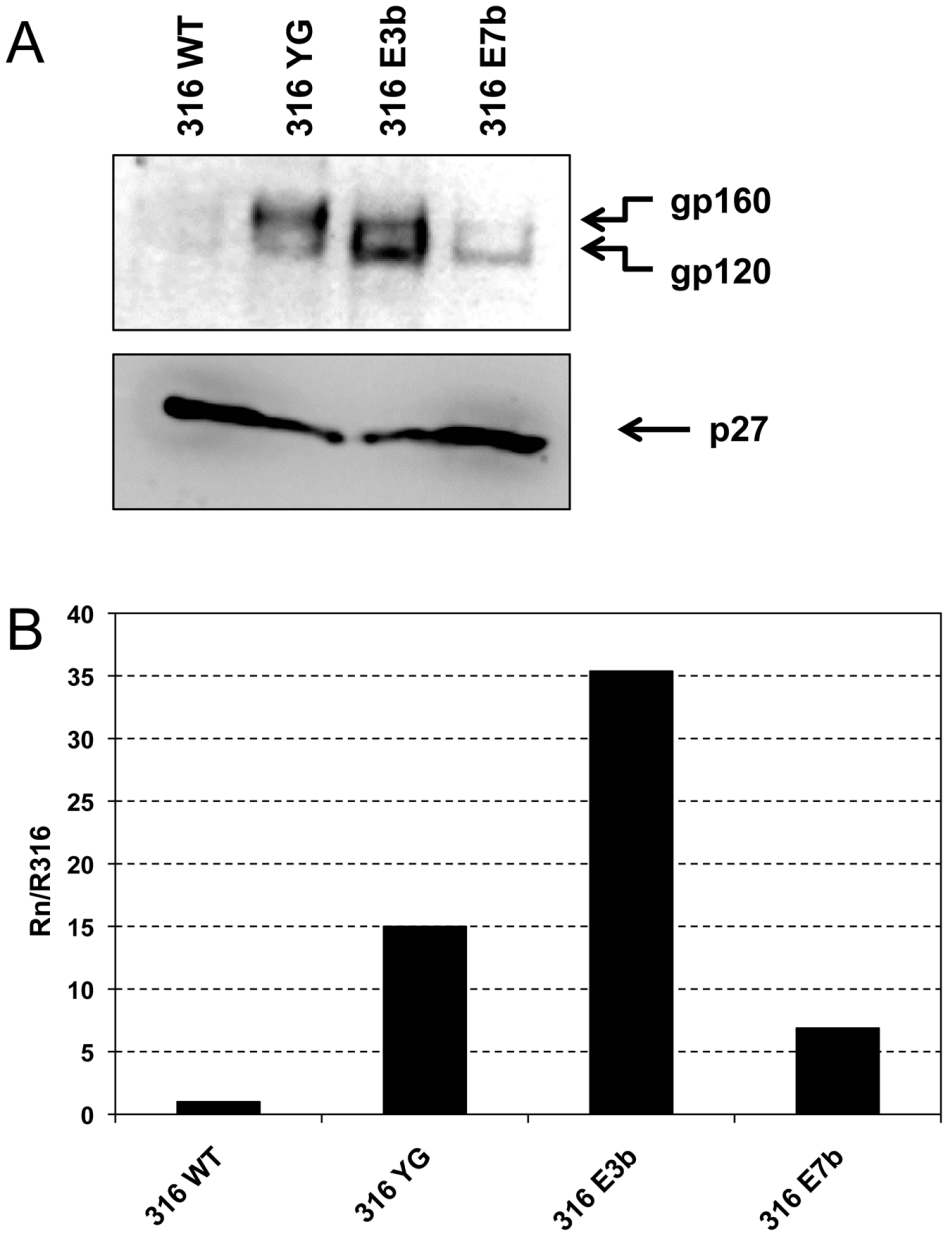
Virion incorporation of SIVmac316 Env is highly responsive to mutations of potential trafficking motifs. Viruses were produced by transfection of HEK293T cells and virions were pelleted from the clarified supernatants. (**A**) gp160/gp120 (top panel) and p27 (bottom panel) were detected by Western blot using antibody 3.11H and 2F12, respectively. (**B**) Relative Env incorporation into virions. The ratios of gp120 to p27 were calculated by phosphorimaging analysis. Data are presented as the gp120:p27 ratio of mutant virions (Rn) relative to the gp120:p27 ratio of SIVmac316 wild-type (WT) virions (R316). Data shown are representative of three independent experiments.

Env encoded by SIVmac316 (Env316) differs from Env of SIVmac239 (Env239) by only eight amino acids, one of which is located within gp41CD, but has been shown to differ considerably with respect to multiple structural and functional properties [31], [36], [42]. In particular, Env316 has been postulated to have a more “open” conformation than its SIVmac239 counterpart resulting in increased neutralization sensitivity [36]. Moreover, truncation of Env316 at amino acid E767 has been shown to increase Env incorporation into virions similar to or slightly less than truncation of Env239; however, the increase in viral infectivity of SIVmac316 resulting from this mutation has been reported as an exceptional 480-fold relative to the wild-type parent strain, compared to a modest 2.5-fold increase for SIVmac239 [39]. Importantly, virion incorporation of Env316 is only half as efficient as Env239 incorporation, and the inherent infectivity of SIVmac316 is about 20-fold lower than the inherent infectivity of SIVmac239 [39]. To gauge the importance of the potential trafficking motifs in gp41CD in the context of an altered structure with similar amino-acid sequence, mutations YG, E3b and E7b (Fig. 1) were introduced into the genetic background of SIVmac316. Virions were produced by transfection of HEK293T cells, pelleted and p27-normalized samples analyzed by Western blot to measure incorporation of Env into virions (Fig. 7). Removal of only the membrane-proximal YXXΦ motif was sufficient to strongly increase Env incorporation into SIVmac316 virions. Interestingly, mutant Env316 YG also incorporated a disproportionately high amount of gp160 into virions, which was not observed with other mutants. Disruption of the first six potential trafficking motifs in gp41CD (mutant E3b) increased Env316 incorporation even more strongly. Consistent with the notion that the C-terminal trafficking motifs may be required for efficient Env incorporation, Env316 mutant E7b was included in virions far less effectively than mutants YG or E3b, but still at higher levels than wild-type Env316. These data further substantiate the observation that the membrane-proximal YXXΦ motif and the central trafficking motifs serve to reduce the level of Env incorporation into virions, whereas the C-terminal motifs seem to contribute to incorporation. It is important to note that, while mutations in Env239 and Env316 affected virion incorporation in a qualitatively similar fashion, Env316 appeared to be far more responsive to mutations of potential trafficking motifs in gp41CD than Env239 and essentially recapitulated the phenotype observed with the E767* mutant (Table 2).

**Table 2:** Comparison of Env239 and Env316 mutations.

	Wild-type Env	Env E767*	Env E3b
**SIVmac239**			
Surface expression	+	++	+
Virion incorporation	+	+++++	++
Infectivity	+	++	+
**SIVmac316**			
Surface expression	+	n.d.	n.d.
Virion incorporation	+	++++	+++++
Infectivity	+	++++++	+++

*Relative to homologous wild-type Env. N.d., not determined.

### Relevance of potential trafficking motifs for viral infectivity of SIVmac316

**Figure 8:**
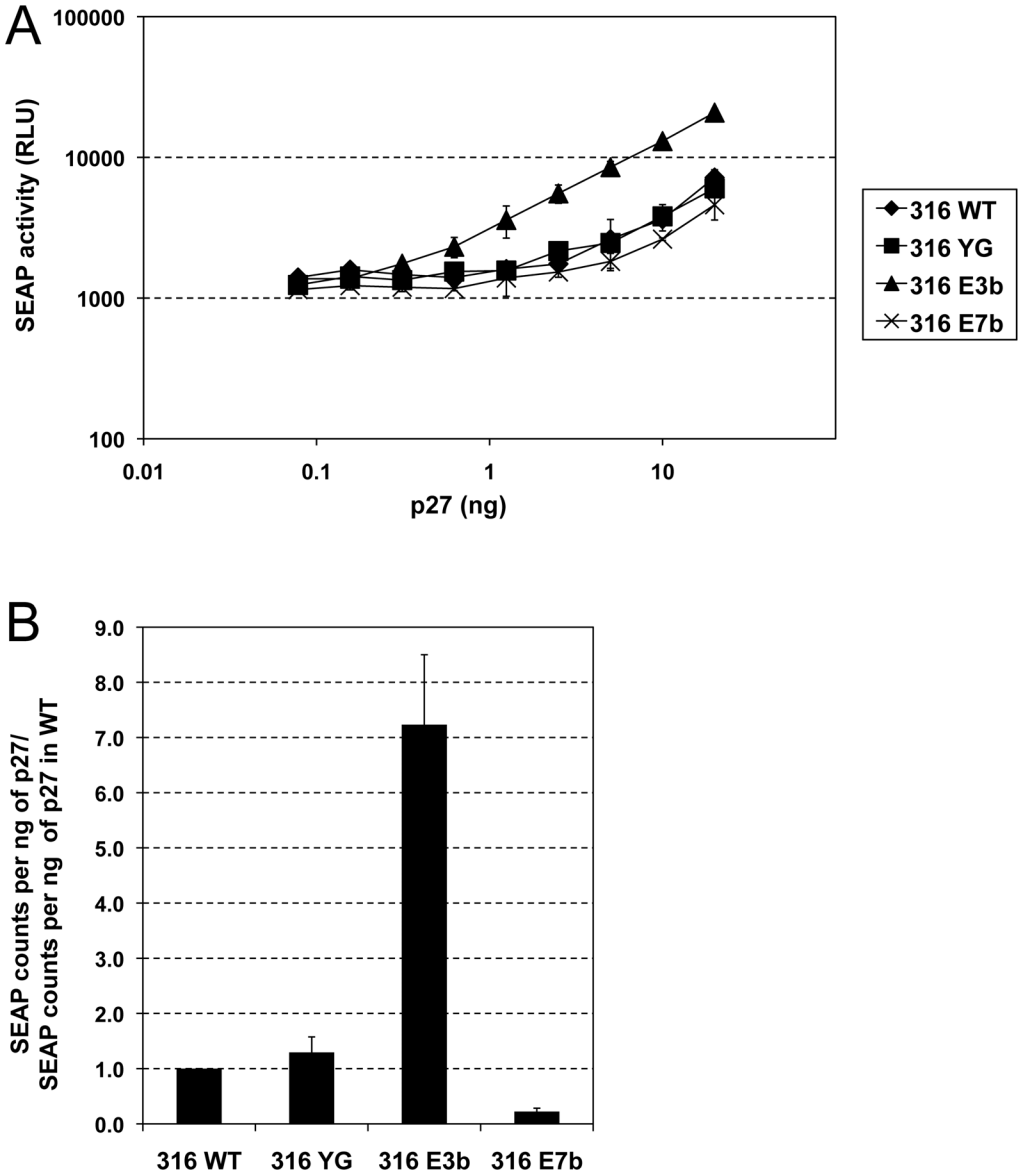
Viral infectivity of SIVmac316 is increased when multiple potential trafficking motifs in gp41CD are eliminated. Viruses were produced by transfection of HEK293T cells and virions were pelleted from the clarified supernatants. Stocks were normalized for p27 content and used to infect C8166-45 SIV-SEAP cells with serial dilutions. (**A**) SEAP activity 3 days after infection. Error bars indicate standard deviation of triplicates. (**B**) SEAP activity per ng of p27 input, normalized for wild-type virus. Average of 2-3 independent experiments, error bars indicate standard deviation. WT, wild-type. SEAP, secreted alkaline phosphatase.

Next, we analyzed the effect of disrupting potential trafficking motifs in gp41CD of SIVmac316 on viral infectivity (Fig. 8 and Table 1). The increase in Env316 incorporation of mutant YG did not translate into a major change in viral infectivity, which may be connected to the high levels of uncleaved and therefore fusion-incompetent gp160 incorporated into virions (Fig. 7A). The SIVmac316 E3b mutant, however, displayed a markedly enhanced infectivity of 7.2-fold over wild-type levels, which likely reflects the drastic increase in Env incorporation (Fig. 7) associated with this mutation in the context of SIVmac316. Infectivity of SIVmac316 mutant E7b was reduced 4.5-fold compared to wild-type infectivity but approximately 33-fold relative to the E3b mutant. Considering that Env316 incorporation of mutant E7b was elevated over wild-type levels, this result further corroborates the notion that the defect in viral infectivity associated with mutation of the C-terminal potential trafficking motifs is likely due to a structural defect independent of Env incorporation, as suggested for SIVmac239. As with regard to Env incorporation, mutations of Env239 and Env316 gp41CD affected viral infectivity with the same tendencies, but the magnitude of the effects was far greater for Env316 (Table 2).

## Discussion

The cytoplasmic domain of SIV gp41 contains twelve sequence motifs with the inherent potential to regulate or modulate intracellular trafficking (Fig. 1). Based on their location, these motifs can be loosely grouped into three categories: (i) the well-characterized membrane-proximal YXXΦ motif; (ii) a central cluster of eight motifs, including two YXXΦ and six dileucine motifs; and (iii) a series of three separate motifs located towards the C terminus, including one YXXΦ, one IL and one LL motif. In this study, we systematically characterized the role of these potential trafficking motifs within the cytoplasmic domain of the SIVmac239 Env glycoprotein with regard to cell-surface expression of Env, incorporation of Env into virions and viral infectivity.

Despite intense research on this topic, it remains incompletely understood how gp41CD achieves highly efficient down-regulation of Env cell-surface expression. While two synergistically acting, endocytotically active sequence motifs have been identified (the membrane-proximal YXXΦ motif, which has been verified as active in HIV-1 and SIV [10]–[12], and the C-terminal dileucine motif, which remains to be confirmed in SIV [13]), mutations of these sequences alone do not typically replicate the strong increase in cell-surface expression of Env observed with truncation of gp41CD C-terminal to the membrane-proximal YXXΦ motif [13]–[15], [23]. To unmask any redundancy between the multiple potential endocytosis motifs, we disrupted all twelve YXXΦ and dileucine motifs in gp41CD in progressive combinations; these mutations did not significantly affect cellular expression levels (Fig. 2). The effect of disrupting the potential trafficking motifs on cell-surface levels of Env239 was minimal and none of the observed increases were statistically significant. However, all mutant Env proteins showed a trend towards slightly elevated cell-surface levels, ranging from an increase of 1.2- to 1.4-fold above wild-type levels (Fig. 3 and Table 1).

Intriguingly, the effects of mutating some of the potential trafficking motifs in gp41CD on SIV Env incorporation into virions and viral infectivity reported here were the direct opposite of phenotypes observed with similar mutations in gp41CD of HIV-1 Env. Specifically, Bhakta *et al*. have demonstrated that disruption of these motifs in progressive combinations in HIV-1 gp41CD considerably reduces both Env incorporation and viral infectivity [24], whereas the same approach in SIVmac239 gp41CD resulted in no change or modest increases with most mutants (Fig. 4, 5, 7, 8 and Table 1). This is somewhat unexpected as gp41CD of HIV-1 and SIV generally maintain a great deal of functional conservation, even in the absence of consistent sequence conservation, although exceptions are not unprecedented [22]. A possible explanation for this discrepancy is provided by a recent report by Qi *et al*., which demonstrated that the cellular protein Rab11-FIP1c is essential for HIV-1 Env incorporation in a gp41CD-dependent manner [43]. Disruption of potential intracellular-trafficking motifs in HIV-1 gp41CD may block the interaction with Rab11-FIP1c, thereby inhibiting HIV-1 Env incorporation into virions. HIV-1 is not capable of incorporating gp41CD-truncated Env in all but a few cell lines, whereas SIV incorporates gp41CD-truncated Env universally [7], [22]. Thus, SIV seems to have evolved a mechanism of Env incorporation that differs from HIV-1 and hence may be independent of Rab11-FIP1c. This may explain why mutations of potential intracellular-trafficking motifs in SIV gp41CD did not negatively affect Env incorporation. However, no data are available on the relevance of Rab11-FIP1c for SIV Env incorporation or even on the interaction motifs in HIV-1 gp41CD that mediate Rab11-FIP1c function, so this explanation remains hypothetical pending further studies.

In this study, HEK293T cells were chosen to produce replication-competent virions as they are easily and efficiently transfected and cannot be infected by the produced virions, preventing mutations in the viral genome and guaranteeing a high level of consistency between independently produced virus stocks. Although we cannot rule out that some of the effects of the mutations reported here may be cell-type-dependent, it seems unlikely considering that virion incorporation of SIV Env has not been reported to be negatively affected by truncation in any cell line.

Disruption of the membrane-proximal YXXΦ motif of SIV gp41CD resulted in a clear increase in Env incorporation into virions above wild-type levels, although this did not translate into an increase in viral infectivity of the corresponding mutants (Table 1). The observation that Env incorporation and viral infectivity correlate poorly and are strain-dependent has been reported before [23], [39], [44]. In the context of strain SIVmac239, truncation at amino acid E767 increases Env content in virions by 25-fold but infectivity by only 2.5-fold [39]. However, the same mutation in SIVmac239-M5 is associated with a 10-fold increase in Env incorporation and a 24-fold increase in infectivity [39]. The most extreme case is that of SIVmac316, in which the E767* mutation yields a 14-fold increase in Env incorporation and an immense 480-fold increase in infectivity [39]. It is not clear why Env incorporation and viral infectivity are only loosely associated. Wild-type SIV and HIV-1 virions incorporate only 7–16 Env trimers per virion [23], [45], [46], or even less [47]. It is conceivable that near-saturation of the virion with Env is required before meaningful increases in infectivity result. More importantly, mutations of gp41CD can affect the inherent fusogenicity of the Env trimer, introducing a confounding variable independent of Env incorporation [39], [41], [44], [48]. The observation that reconstituting the membrane-proximal YXXΦ motif in the background of mutant E3b led to a reduction in Env incorporation into virions but a concomitant increase in viral infectivity provides further evidence that this motif is structurally important for efficient viral entry (Fig. 6).

The central cluster of potential trafficking motifs appeared to mildly enhance the effects of the membrane-proximal YXXΦ motif with respect to virion incorporation of Env239. Mutant E4b, which is lacking the membrane-proximal YXXΦ motif and the entire central cluster, incorporated Env239 at 8.2-fold higher levels than wild-type virus, thus doubling the effect of the YG mutation (Fig. 4 and Table 1). The C-terminal half of the central cluster is contained within the lentivirus lytic peptide 2 (LLP-2) region [22]. This is of note, as Bhakta *et al*. observed the largest decrease in Env incorporation and infectivity in mutants in which potential trafficking motifs within the LLP-2 region were disrupted [24], emphasizing the direct contrast between gp41CD of SIV and HIV-1 in this particular regard.

Extension of the mutations to include the three C-terminal sequence motifs resulted in a stepwise reduction of both Env incorporation and viral infectivity, while cell-surface expression remained unaffected (Fig. 3, 4, 5 and Table 1). Our results are consistent with the observation by Manrique *et al*. that short C-terminal deletions of SIV gp41CD of 20 amino acids, which includes the residues changed in mutants E6b and E7b, are sufficient to drastically reduce Env incorporation and viral infectivity [49]. It is possible that the interaction of gp41CD with the matrix (MA) component of the Gag precursor protein is interrupted by mutations E5b - E7b. There are multiple reports demonstrating that mutations in gp41CD which block Env incorporation can be compensated for by mutations in MA and truncation of gp41CD can counteract MA mutations which block Env incorporation [7], [50]–[52], suggesting that the conformations of gp41CD and MA need to be compatible to allow efficient Env incorporation [53]. It is interesting to note that mutant E6b still retained 93% of Env239 incorporation compared to wild-type (a reduction compared to mutants of the central cluster) but exhibited substantially reduced infectivity. This implies that mutated Env protein that is indeed incorporated successfully into virions is no longer fully functional.

In direct comparison of the closely related strains SIVmac239 and SIVmac316, whose Env proteins differ by only eight amino acids, SIVmac316 was markedly more responsive to the effects of mutations YG, E3b and E7b (Table 2). To interpret these data accurately, it is important to note that SIVmac316 incorporates only about half as many Env trimers per virion as SIVmac239 and has an inherent infectivity that is about 20-fold lower than the inherent infectivity of SIVmac239 [39], despite the close sequence similarity between the respective Env proteins. With such a low baseline it is likely that even small changes may result in major shifts in phenotype. Furthermore, it has been postulated that Env316 has a more “open” conformation than Env239 [36]. Neutralization sensitivity, low infectivity in standard cell-culture systems, and a more open Env conformation likely emerged in SIVmac316 as a result of selection for replication in macrophages with little or no CD4 on their surface [31], [36], [39], [42], [54]. The observed hypersensitivity of Env316 to the mutations analyzed in this study is consistent with the notion that the looser conformation not only applies to the extracellular domains of Env316 but also to gp41CD, making it more accessible to proteins of the cellular trafficking machinery. Thus the reason that mutations of Env316 but not of Env239 recapitulate the phenotype of the E767* truncation with respect to virion incorporation may be that the potential trafficking motifs are not accessible in Env239 due to its tighter conformation, but accessible and therefore functional in Env316. However, it is important to note that there is currently no information available about the actual conformation of gp41CD in the context of the full Env trimer, and further studies will be required to determine if this conformation does indeed differ between Env from different strains.

The observation that none of the Env239 amino-acid mutants investigated in this study were able to reproduce the phenotype of truncation at E767 demonstrates that the major increases in cell-surface expression and virion incorporation observed upon Env239 truncation do not result from the loss of the known consensus motifs. An alternative explanation for the effects of the E767* mutation in the background of SIVmac239 is provided by the observation that this truncation drastically increases Env protein levels in cells, as reported here (Fig. 2) and by others [55]–[59]. Thus, the effects of gp41CD truncation may simply be caused by the higher levels of Env protein in the cell. Further studies of this phenomenon are required to elucidate the molecular mechanism underlying this Env enrichment and its relevance for cell-surface expression, Env incorporation into virions and viral infectivity. While we favor the hypothesis that the effects of Env239 E767* truncation result directly from increased Env levels within the cell, there are several other possibilities, which are not mutually exclusive. For instance, intracellular trafficking is not limited to YXXΦ and dileucine motifs, but is also regulated by more complex determinants such as ubiquitination, acidic clusters, cargo-specific adaptors, and non-canonical sequence motifs [20], [26], [60], which were not addressed in this study. Furthermore, the unusual length of gp41CD itself may be a determining factor for efficiency of progression through the secretory pathway, virion incorporation, endocytosis and/or recycling after endocytosis.

## Conclusions

In this study, we have demonstrated that potential trafficking motifs in gp41CD of SIVmac239 have only limited functionality and are not sufficient to explain the effects of gp41CD truncation at E767. Interestingly, however, we found that eliminating the very same motifs in Env316 did have a considerable effect on virion incorporation and viral infectivity, implying that the motifs may indeed be functional but inaccessible in the tighter conformation of Env239. The results presented here contrast sharply with observations made for gp41CD of HIV-1, for which loss of potential trafficking motifs in gp41CD causes decreases in virion incorporation and infectivity [24], whereas we detected no changes or moderate increases in response to such mutations in SIVmac239.

From an evolutionary perspective, the low levels of Env surface expression and Env incorporation into virions that appear to be partially mediated by the membrane-proximal YXXΦ motif and possibly the central cluster of potential trafficking motifs may benefit the virus by reducing the number of targets for antibody-mediated neutralization. Considering the very large increase of Env trimers per virion that seems to be necessary to effect even modest increases in viral infectivity (Table 1), this is likely to be a highly favorable trade-off during infection, which might explain the apparent redundancy of sequence motifs in gp41CD capable of modulating Env incorporation into virions.

## References

[1] Allan JS, Coligan JE, Barin F, McLane MF, Sodroski JG, et al (1985) Major Glycoprotein Antigens that Induce Antibodies in AIDS Patients are Encoded by HTLV-III. Science 228:1091–1094. 2986290 10.1126/science.2986290

[2] Veronese FD, DeVico AL, Copeland TD, Oroszlan S, Gallo RC, et al (1985) Characterization of gp41 as the Transmembrane Protein Coded by the HTLV-III/LAV Envelope Gene. Science 229:1402–1405. 2994223 10.1126/science.2994223

[3] Veronese FD, Joseph B, Copeland TD, Oroszlan S, Gallo RC, et al (1989) Identification of simian immunodeficiency virus SIVMAC env gene products. J Virol 63:1416–1419. 2464704 10.1128/jvi.63.3.1416-1419.1989 PMC247843

[4] Hallenberger S, Bosch V, Angliker H, Shaw E, Klenk HD, et al (1992) Inhibition of furin-mediated cleavage activation of HIV-1 glycoprotein gp160. Nature 360:358–361 doi:10.1038/360358a0. 1360148

[5] Hallenberger S, Moulard M, Sordel M, Klenk HD, Garten W (1997) The role of eukaryotic subtilisin-like endoproteases for the activation of human immunodeficiency virus glycoproteins in natural host cells. J Virol 71:1036–1045. 8995623 10.1128/jvi.71.2.1036-1045.1997 PMC191154

[6] Moulard M, Decroly E (2000) Maturation of HIV envelope glycoprotein precursors by cellular endoproteases. Biochim Biophys Acta 1469:121–132. 11063880 10.1016/s0304-4157(00)00014-9

[7] Checkley MA, Luttge BG, Freed EO (2011) HIV-1 envelope glycoprotein biosynthesis, trafficking, and incorporation. J Mol Biol 410:582–608 doi:10.1016/j.jmb.2011.04.042. 21762802 PMC3139147

[8] Kowalski M, Potz J, Basiripour L, Dorfman T, Goh WC, et al (1987) Functional regions of the envelope glycoprotein of human immunodeficiency virus type 1. Science 237:1351–1355. 3629244 10.1126/science.3629244

[9] Ohno H, Aguilar RC, Fournier M-C, Hennecke S, Cosson P, et al (1997) Interaction of Endocytic Signals from the HIV-1 Envelope Glycoprotein Complex with Members of the Adaptor Medium Chain Family. Virology 238:305–315. 9400603 10.1006/viro.1997.8839

[10] LaBranche CC, Sauter MM, Haggarty BS, Vance PJ, Romano J, et al (1995) A single amino acid change in the cytoplasmic domain of the simian immunodeficiency virus transmembrane molecule increases envelope glycoprotein expression on infected cells. J Virol 69:5217–5227. 7636963 10.1128/jvi.69.9.5217-5227.1995 PMC189351

[11] Rowell JF, Stanhope PE, Siliciano RF (1995) Endocytosis of Endogenously Synthesized HIV-1 Envelope Protein. J Immunol 155:473–488. 7602119

[12] Sauter MM, Pelchen-Matthews A, Bron R, Marsh M, LaBranche CC, et al (1996) An internalization signal in the simian immunodeficiency virus transmembrane protein cytoplasmic domain modulates expression of envelope glycoproteins on the cell surface. J Cell Biol 132:795–811. 8603913 10.1083/jcb.132.5.795 PMC2120738

[13] Byland R, Vance PJ, Hoxie JA, Marsh M (2007) A Conserved Dileucine Motif Mediates Clathrin and AP-2-dependent Endocytosis of the HIV-1 Envelope Protein. Mol Biol Cell 18:414–425. 17108326 10.1091/mbc.E06-06-0535 PMC1783771

[14] Berlioz-Torrent C, Shacklett BL, Erdtmann L, Delamarre L, Bouchaert I, et al (1999) Interactions of the Cytoplasmic Domains of Human and Simian Retroviral Transmembrane Proteins with Components of the Clathrin Adaptor Complexes Modulate Intracellular and Cell Surface Expression of Envelope Glycoproteins. J Virol 73:1350–1361. 9882340 10.1128/jvi.73.2.1350-1361.1999 PMC103959

[15] Bowers K, Pelchen-Matthews A, Hoening S, Vance PJ, Creary L, et al (2000) The Simian Immunodeficiency Virus Envelope Glycoprotein Contains Multiple Signals that Regulate its Cell Surface Expression and Endocytosis. Traffic 1:661–674. 11208154 10.1034/j.1600-0854.2000.010810.x

[16] Boge M, Wyss S, Bonifacino JS, Thali M (1998) A Membrane-proximal Tyrosine-based Signal Mediates Internalization of the HIV-1 Envelope Glycoprotein via Interaction with the AP-2 Clathrin Adaptor. J Biol Chem 273:15773–15778. 9624176 10.1074/jbc.273.25.15773

[17] Fultz PN, Vance PJ, Endres MJ, Tao B, Dvorin JD, et al (2001) In vivo attenuation of simian immunodeficiency virus by disruption of a tyrosine-dependent sorting signal in the envelope glycoprotein cytoplasmic tail. J Virol 75:278–291 doi:10.1128/JVI.75.1.278-291.2001. 11119598 PMC113922

[18] Breed MW, Jordan APO, Aye PP, Lichtveld CF, Midkiff C, et al (2012) Loss of a Tyrosine-Dependent Trafficking Motif in the Simian Immunodeficiency Virus Envelope Cytoplasmic Tail Spares Mucosal CD4 Cells but Does Not Prevent Disease Progression. J Virol. 10.1128/JVI.01928-12 PMC3554169 23152518

[19] Traub LM (2009) Tickets to ride: selecting cargo for clathrin-regulated internalization. Nat Rev Mol Cell Biol 10:583–596 doi:10.1038/nrm2751. 19696796

[20] Bonifacino JS, Traub LM (2003) Signals for sorting of transmembrane proteins to endosomes and lysosomes. Annu Rev Biochem 72:395–447 doi:10.1146/annurev.biochem.72.121801.161800. 12651740

[21] Earl PL, Moss B, Doms RW (1991) Folding, interaction with GRP78-BiP, assembly, and transport of the human immunodeficiency virus type 1 envelope protein. J Virol 65:2047–2055. 1900540 10.1128/jvi.65.4.2047-2055.1991 PMC240054

[22] Postler TS, Desrosiers RC (2012) The Tale of the Long Tail: the Cytoplasmic Domain of HIV-1 gp41. J Virol. 10.1128/JVI.02053-12 PMC3536369 23077317

[23] Yuste E, Reeves JD, Doms RW, Desrosiers RC (2004) Modulation of Env Content in Virions of Simian Immunodeficiency Virus: Correlation with Cell Surface Expression and Virion Infectivity. J Virol 78:6775–6785. 15194752 10.1128/JVI.78.13.6775-6785.2004 PMC421677

[24] Bhakta SJ, Shang L, Prince JL, Claiborne DT, Hunter E (2011) Mutagenesis of tyrosine and di-leucine motifs in the HIV-1 envelope cytoplasmic domain results in a loss of Env-mediated fusion and infectivity. Retrovirology 8:37 doi:10.1186/1742-4690-8-37. 21569545 PMC3117779

[25] Wyss S, Berlioz-Torrent C, Boge M, Blot G, Hoening S, et al (2001) The Highly Conserved C-Terminal Dileucine Motif in the Cytosolic Domain of the Human Immunodeficiency Virus Type 1 Envelope Glycoprotein Is Critical for Its Association with the AP-1 Clathrin Adaptor. J Virol 75:2982–2992. 11222723 10.1128/JVI.75.6.2982-2992.2001 PMC115924

[26] Robinson MS (2004) Adaptable adaptors for coated vesicles. Trends Cell Biol 14:167–174 doi:10.1016/j.tcb.2004.02.002. 15066634

[27] Lodge R, Lalonde J-P, Lemay G, Cohen EA (1997) The membrane-proximal intracytoplasmic tyrosine residue of HIV-1 envelope glycoprotein is critical for basolateral targeting of viral budding in MDCK cells. EMBO J 16:695–705. 9049299 10.1093/emboj/16.4.695 PMC1169671

[28] Deschambeault J, Lalonde JP, Cervantes-Acosta G, Lodge R, Cohen EA, et al (1999) Polarized human immunodeficiency virus budding in lymphocytes involves a tyrosine-based signal and favors cell-to-cell viral transmission. J Virol 73:5010–5017. 10233963 10.1128/jvi.73.6.5010-5017.1999 PMC112545

[29] Chen BK (2012) T cell virological synapses and HIV-1 pathogenesis. Immunol Res. 10.1007/s12026-012-8320-8 22477441

[30] Kuiken C, Leitner T, Foley B, Hahn B, Marx P, et al (2009) HIV Sequence Compendium 2009. Kuiken C, Leitner T, Foley B, Hahn B, Marx Pet al., editors Theoretical Biology and Biophysics Group, Los Alamos National Laboratory, NM, LA-UR 09-03280.

[31] Mori K, Ringler DJ, Kodama T, Desrosiers RC (1992) Complex determinants of macrophage tropism in env of simian immunodeficiency virus. J Virol 66:2067–2075. 1548752 10.1128/jvi.66.4.2067-2075.1992 PMC288997

[32] Gibbs JS, Regier DA, Desrosiers RC (1994) Construction and in vitro properties of SIVmac mutants with deletions in “nonessential” genes. AIDS Res Hum Retroviruses 10:607–616. 7917522 10.1089/aid.1994.10.607

[33] Regier DA, Desrosiers RC (1990) The complete nucleotide sequence of a pathogenic molecular clone of simian immunodeficiency virus. AIDS Res Hum Retroviruses 6:1221–1231. 2078405 10.1089/aid.1990.6.1221

[34] Marcon L, Sodroski J (1997) High degree of sensitivity of the simian immunodeficiency virus (SIVmac) envelope glycoprotein subunit association to amino acid changes in the glycoprotein 41 ectodomain. AIDS Res Hum Retroviruses 13:441–447. 9100985 10.1089/aid.1997.13.441

[35] Means RE, Greenough T, Desrosiers RC (1997) Neutralization sensitivity of cell culture-passaged simian immunodeficiency virus. J Virol 71:7895–7902. 9311879 10.1128/jvi.71.10.7895-7902.1997 PMC192146

[36] Johnson WE, Sanford H, Schwall L, Burton DR, Parren PW, et al (2003) Assorted mutations in the envelope gene of simian immunodeficiency virus lead to loss of neutralization resistance against antibodies representing a broad spectrum of specificities. J Virol 77:9993–10003. 12941910 10.1128/JVI.77.18.9993-10003.2003 PMC224602

[37] Cole KS, Alvarez M, Elliott DH, Lam H, Martin E, et al (2001) Characterization of neutralization epitopes of simian immunodeficiency virus (SIV) recognized by rhesus monoclonal antibodies derived from monkeys infected with an attenuated SIV strain. Virology 290:59–73. 11883006 10.1006/viro.2001.1144

[38] Higgins JR, Sutjipto S, Marx PA, Pedersen NC (1992) Shared antigenic epitopes of the major core proteins of human and simian immunodeficiency virus isolates. J Med Primatol 21:265–269. 1383547

[39] Yuste E, Johnson W, Pavlakis GN, Desrosiers RC (2005) Virion Envelope Content, Infectivity, and Neutralization Sensitivity of Simian Immunodeficiency Virus. J Virol 79:12455–12463. 16160173 10.1128/JVI.79.19.12455-12463.2005 PMC1211544

[40] Roeth JF, Collins KL (2006) Human immunodeficiency virus type 1 Nef: adapting to intracellular trafficking pathways. Microbiol Mol Biol Rev 70:548–563 doi:10.1128/MMBR.00042-05. 16760313 PMC1489538

[41] Day JR, Muenk C, Guatelli JC (2004) The Membrane-Proximal Tyrosine-Based Sorting Signal of Human Immunodeficiency Virus Type 1 gp41 Is Required for Optimal Viral Infectivity. J Virol 78:1069–1079. 14722262 10.1128/JVI.78.3.1069-1079.2004 PMC321364

[42] Puffer BA, Pöhlmann S, Edinger AL, Carlin D, Sanchez MD, et al (2002) CD4 independence of simian immunodeficiency virus Envs is associated with macrophage tropism, neutralization sensitivity, and attenuated pathogenicity. J Virol 76:2595–2605. 11861825 10.1128/JVI.76.6.2595-2605.2002 PMC135960

[43] Qi M, Williams JA, Chu H, Chen X, Wang J-J, et al (2013) Rab11-FIP1C and Rab14 direct plasma membrane sorting and particle incorporation of the HIV-1 envelope glycoprotein complex. PLoS Pathog 9:e1003278 doi:10.1371/journal.ppat.1003278. 23592992 PMC3616983

[44] West JT, Weldon SK, Wyss S, Lin X, Yu Q, et al (2002) Mutation of the dominant endocytosis motif in human immunodeficiency virus type 1 gp41 can complement matrix mutations without increasing Env incorporation. J Virol 76:3338–3349. 11884559 10.1128/JVI.76.7.3338-3349.2002 PMC136014

[45] Zhu P, Chertova E, Bess JJ, Lifson JD, Arthur LO, et al (2003) Electron tomography analysis of envelope glycoprotein trimers on HIV and simian immunodeficiency virus virions. Proc Natl Acad Sci USA 100:15812–15817. 14668432 10.1073/pnas.2634931100 PMC307650

[46] Chertova E, Bess JWJ, Crise BJ, Sowder IR, Schaden TM, et al (2002) Envelope glycoprotein incorporation, not shedding of surface envelope glycoprotein (gp120/SU), Is the primary determinant of SU content of purified human immunodeficiency virus type 1 and simian immunodeficiency virus. J Virol 76:5315–5325. 11991960 10.1128/JVI.76.11.5315-5325.2002 PMC137021

[47] Del Prete GQ, Scarlotta M, Newman L, Reid C, Parodi LM, et al (2013) Comparative characterization of transfection- and infection-derived simian immunodeficiency virus challenge stocks for in vivo nonhuman primate studies. J Virol 87:4584–4595 doi:10.1128/JVI.03507-12. 23408608 PMC3624367

[48] Kalia V, Sarkar S, Gupta P, Montelaro RC (2003) Rational site-directed mutations of the LLP-1 and LLP-2 lentivirus lytic peptide domains in the intracytoplasmic tail of human immunodeficiency virus type 1 gp41 indicate common functions in cell-cell fusion but distinct roles in virion envelope incorporation. J Virol 77:3634–3646. 12610139 10.1128/JVI.77.6.3634-3646.2003 PMC149489

[49] Manrique JM, Celma CC, Affranchino JL, Hunter E, Gonzalez SA (2001) Small variations in the length of the cytoplasmic domain of the simian immunodeficiency virus transmembrane protein drastically affect envelope incorporation and virus entry. AIDS Res Hum Retroviruses 17:1615–1624. 11779349 10.1089/088922201753342022

[50] Mammano F, Kondo E, Sodroski J, Bukovsky A, Göttlinger HG (1995) Rescue of human immunodeficiency virus type 1 matrix protein mutants by envelope glycoproteins with short cytoplasmic domains. J Virol 69:3824–3830. 7745730 10.1128/jvi.69.6.3824-3830.1995 PMC189100

[51] Murakami T, Freed EO (2000) Genetic evidence for an interaction between human immunodeficiency virus type 1 matrix and alpha-helix 2 of the gp41 cytoplasmic tail. J Virol 74:3548–3554. 10729129 10.1128/jvi.74.8.3548-3554.2000 PMC111863

[52] Freed EO, Martin MA (1995) Virion incorporation of envelope glycoproteins with long but not short cytoplasmic tails is blocked by specific, single amino acid substitutions in the human immunodeficiency virus type 1 matrix. J Virol 69:1984–1989. 7853546 10.1128/jvi.69.3.1984-1989.1995 PMC188822

[53] Tedbury PR, Ablan SD, Freed EO (2013) Global Rescue of Defects in HIV-1 Envelope Glycoprotein Incorporation: Implications for Matrix Structure. PLoS Pathog 9:e1003739 doi:10.1371/journal.ppat.1003739. 24244165 PMC3828165

[54] Mori K, Rosenzweig M, Desrosiers RC (2000) Mechanisms for adaptation of simian immunodeficiency virus to replication in alveolar macrophages. J Virol 74:10852–10859. 11044136 10.1128/jvi.74.22.10852-10859.2000 PMC110966

[55] Hirsch VM, Edmondson P, Murphey-Corb M, Arbeille B, Johnson PR, et al (1989) SIV adaptation to human cells. Nature 341:573–574 doi:10.1038/341573a0. 2677749

[56] Zingler K, Littman DR (1993) Truncation of the cytoplasmic domain of the simian immunodeficiency virus envelope glycoprotein increases env incorporation into particles and fusogenicity and infectivity. J Virol 67:2824–2831. 8474176 10.1128/jvi.67.5.2824-2831.1993 PMC237607

[57] Spies CP, Compans RW (1994) Effects of cytoplasmic domain length on cell surface expression and syncytium-forming capacity of the simian immunodeficiency virus envelope glycoprotein. Virology 203:8–19. 8030287 10.1006/viro.1994.1449

[58] Spies CP, Ritter GD, Mulligan MJ, Compans RW (1994) Truncation of the cytoplasmic domain of the simian immunodeficiency virus envelope glycoprotein alters the conformation of the external domain. J Virol 68:585–591. 8289362 10.1128/jvi.68.2.585-591.1994 PMC236490

[59] Ritter GDJ, Mulligan MJ, Lydy SL, Compans RW (1993) Cell fusion activity of the simian immunodeficiency virus envelope protein is modulated by the intracytoplasmic domain. Virology 197:255–264. 8212561 10.1006/viro.1993.1586

[60] Kozik P, Francis RW, Seaman MNJ, Robinson MS (2010) A screen for endocytic motifs. Traffic 11:843–855 doi:10.1111/j.1600-0854.2010.01056.x. 20214754 PMC2882754

